# Shooting the messenger: RNA-targetting CRISPR-Cas systems

**DOI:** 10.1042/BSR20170788

**Published:** 2018-06-21

**Authors:** Yifan Zhu, Sanne E. Klompe, Marnix Vlot, John van der Oost, Raymond H.J. Staals

**Affiliations:** Laboratory of Microbiology, Department of Agrotechnology and Food Sciences, Wageningen University, Wageningen 6708 WE, The Netherlands

**Keywords:** bacteriophages, CRISPR, RNA interference, RNA

## Abstract

Since the discovery of CRISPR-Cas (Clustered Regularly Interspaced Short Palindromic Repeats, CRISPR-associated genes) immune systems, astonishing progress has been made on revealing their mechanistic foundations. Due to the immense potential as genome engineering tools, research has mainly focussed on a subset of Cas nucleases that target DNA. In addition, however, distinct types of RNA-targetting CRISPR-Cas systems have been identified. The focus of this review will be on the interference mechanisms of the RNA targetting type III and type VI CRISPR-Cas systems, their biological relevance and their potential for applications.

## Introduction to CRISPR-Cas

CRISPR-Cas (Clustered Regularly Interspaced Short Palindromic Repeats, CRISPR-associated genes) is a prokaryotic adaptive immune system that is present in approximately half of the currently available bacterial and archaeal genomes. It provides sequence-specific defense against mobile genetic elements (MGEs) such as bacteriophages and conjugative plasmids, and plays a role in host gene regulation [1–6].

The mechanism of adaptive immunity by CRISPR-Cas systems can be divided into three different phases: adaptation, expression/processing, and interference. The defense is initiated by a process called ‘adaptation’ or ‘spacer acquisition’ during which short, MGE-derived DNA sequences, called spacers, are stored in repetitive loci on the host chromosome. The spacers are separated by repetitive DNA sequences called repeats, collectively forming the CRISPR array. These CRISPRs function as a genetic memory storage that allows the host to recognize future invasions by previously encountered MGEs. In the second phase, a CRISPR array is transcribed into a long RNA transcript that is subsequently processed into multiple mature CRISPR RNAs (crRNAs) [7–9]. In addition, Cas proteins are encoded by *cas* genes that are generally located in close proximity to the CRISPR array. Together the crRNA and Cas protein(s) form a ribonucleoprotein (RNP) complex that will patrol the cell. In the interference phase, these RNPs use complementarity to the crRNA to detect recurring nucleic acid invaders and utilize the nuclease activity of the Cas proteins to degrade the invading nucleic acids [10].

A very broad range of CRISPR-Cas systems has been discovered to date, resulting in a classification system and a common nomenclature for the associated *cas* genes. The latest classification divides these systems into two classes [11–13], each of which are further divided into three different types and numerous subtypes based on signature *cas* genes. Class 1 systems (encompassing type I, III, and the putative type IV systems) utilize multisubunit RNP complexes, while class 2 systems (encompassing the type II, V, and VI systems) utilize single protein RNP complexes.

**Figure 1:**
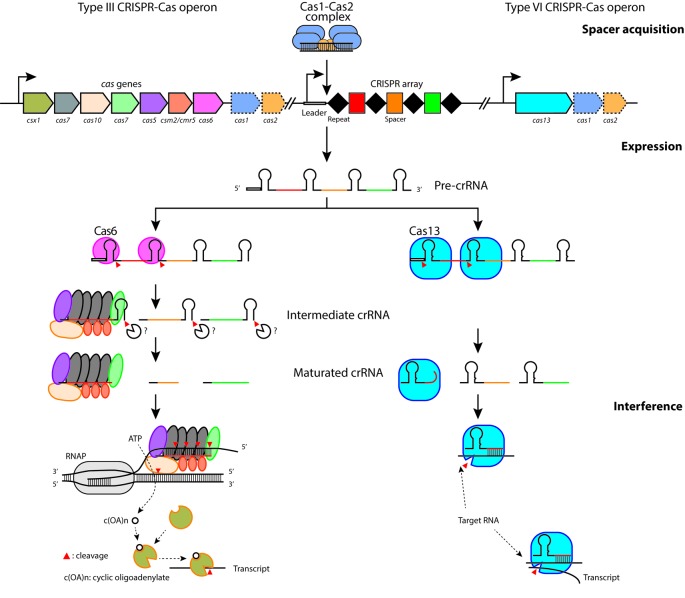
Overview of the three phases of type III and type VI CRISPR-Cas systems Spacer acquisition, upon entry of an MGE (in this case, a bacteriophage genome), the Cas1 (teal) and Cas2 (orange) proteins select and process a region on the invading DNA to generate a spacer, which will be integrated at the leader-first repeat junction of the CRISPR array, consisting of repeats (black diamonds) and pre-existing spacers (multiple colors). Expression, the CRISPR locus is transcribed into a long pre-crRNA transcript. The Cas6 endoribonuclease (light pink) and Cas13 (light blue) proteins cleave the pre-crRNA at fixed positions down- (Cas6) or upstream (Cas13) of the stem-loop structure (formed by the palindromic nature of the repeats). In type III systems, the 3′ end of the intermediate crRNA is further processed by an unknown mechanism. The mature crRNAs assemble with Cas protein(s) to form a functional RNP complex. Interference, the RNP complex scans transcripts for a complementary RNA target, after which the RNA target is degraded by the Cas7 nuclease (in type III) or Cas13 (in type VI). In type III systems, base pairing between the crRNA and the target RNA will activate the HD and palm domain of Cas10 for ssDNA cleavage and cyclic oligoadenylates c(OA)s biosynthesis, respectively. c(OA)s will play a role as a second messenger to trigger the sequence non-specific RNA cleavage activity of Csx1 (olive). In type VI systems, sequence non-specific RNA cleavage is conferred by the higher eukaryotes and prokaryotes nucleotide-binding (HEPN) domain in Cas13 after target RNA binding.

Even though every CRISPR-Cas system has its own characteristics, most rely on direct targetting of invading DNA. Two CRISPR-Cas types, however, have been shown to differ from this standard by using RNA as their *bona fide* target (Figure 1). Many recent reviews have focussed on DNA targetting CRISPR-Cas systems. Instead, this review will discuss the state of the art of the two RNA-targetting CRISPR-Cas types, type III and VI. Their interference mechanisms and fundamental biology will be discussed.

## CRISPR-Cas type III systems

**Figure 2:**
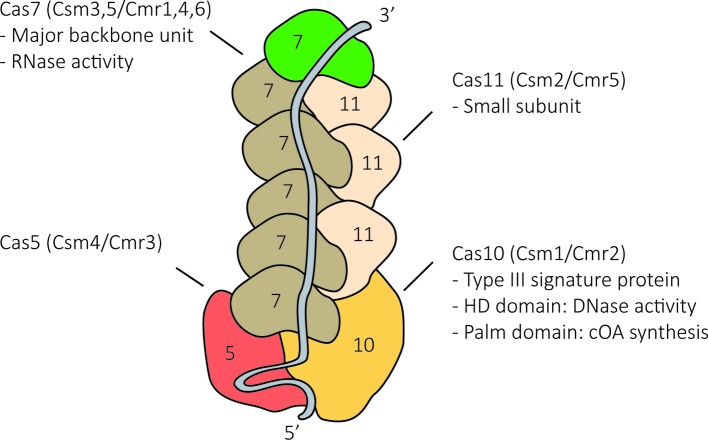
Structural arrangement of a typical type III CRISPR–Cas complex Homologous subunits are depicted by the same color (old nomenclature is mentioned in brackets).

Type III systems belong to the class 1 CRISPR-Cas systems, and as such, their RNP complexes are composed of four–six different Cas proteins (Figure 2). Even though there are subtype-specific variations between different type III RNP complexes, the general structure is largely conserved. The general structure comprises two intertwined helical protein filaments, the backbone of which is formed by multiple copies of the Cas7 protein and multiple copies of the small subunit Cas11 (Csm2 or Cmr5) [14–17]. The signature protein Cas10, together with Cas5, caps one end of the RNP, whereas different variants of the Cas7 protein cap the other end of the structure. In addition, the RNP contains a mature crRNA that comprises a general repeat-derived sequence called the 5′-handle and an unique spacer-derived sequence. The 5′-handle is tightly anchored within the Cas10/Cas5 cap while the spacer-derived sequence of the crRNA spans the backbone of the RNP [18–22].

Type III CRISPR-Cas systems are divided into four subtypes (subtype III-A to III-D). Subtypes III-A and III-B (Csm and Cmr) have been described first, while the less frequently occurring variant subtypes III-C and III-D systems have subsequently been discovered. The signature subunit Cas10 typically contains an HD-type nuclease domain and a GGDD-type cyclase domain (referred to as the palm domain). The amino acid sequence of the Cas10 subunit of subtype III-C differs substantially from that of the other type III subtypes, probably reflecting an inactive cyclase-like domain [12]. Subtype III-D systems usually harbor a Cas10 that lacks the HD domain.

## Spacer adaptation in type III systems

Cas1 and Cas2 proteins are widely conserved amongst CRISPR-Cas systems and were found to be essential for spacer acquisition [23]. Surprisingly, not all type III systems harbor these genes [12]. The lack of these genes is likely complemented by other CRISPR-Cas systems present in the genome. As such, type III systems are expected to use Cas1 and Cas2 from other systems *in trans* and have been shown to utilize mature crRNAs derived from other CRISPR arrays [11,12,24].

A well-conserved feature of all CRISPR-Cas systems is that a complex of Cas1 and Cas2, sometimes complemented with additional proteins, is responsible for the selection of protospacers in invading genomes and the subsequent integration as new spacers in the CRISPR array in the host chromosome. A unique additional mechanism for spacer acquisition by some type III systems was recently discovered. In these specific systems, Cas1 was found to be fused to a reverse transcriptase domain (RT) [25,26]. Since RT enzymes can generate DNA from an RNA template, an RT-Cas1 fusion suggested a possible function for spacer adaptation from RNA. Indeed, this fusion has recently been demonstrated to be capable of spacer acquisition from RNA while retaining its ability to acquire spacers from DNA [25].

## Nuclease activities of type III systems

Currently, type III systems are the only CRISPR-Cas systems known to utilize three different nuclease activities for invader neutralization. For many years, various targets have been identified for different CRISPR-Cas type III subtypes. Initial *in vivo* experiments with the type III-A Csm–crRNA complex of *Staphylococcus epidermidis* indicated that immunity relied on the degradation of target DNA [3]. This was quickly followed-up with *in vitro* experiments with the Cmr complex of the type III-B Cmr in *Pyrococcus furiosus*, which revealed that not DNA but RNA was specifically degraded [27]. This apparent conundrum came to an end when it became clear that a general type III mechanism is based on transcription-dependent (specific RNA binding and cleavage) and subsequent (non-specific) targetting of DNA [28,29]. Interestingly, additional non-specific RNase activity by type III systems was recently demonstrated to occur through allosteric activation of a hyperactive Cas ribonuclease [30,31]. In this section, these three individual nuclease activities will be discussed.

### Specific RNA cleavage

The only sequence-specific degradation facilitated by type III systems is that of the target RNA (containing the protospacer sequence complementary to the crRNA guide). Binding of the target RNA to the crRNA of the effector complex positions the target along the Cas7 subunits forming the RNP backbone [16,32,33]. Structural data revealed that a conserved β-hairpin present in Cas7 (Csm3 or Cmr4) disrupts the RNA duplex at 6-nt intervals [34]. This steric conflict results in the outward ‘flipping’ of nucleotides, positioning them for cleavage facilitated by a conserved aspartate residue which is also present in Cas7 [16,34]. Since Cas7 is present in multiple copies, this results in multiple cleavage sites within the targetted RNA sequences. In accordance with the RNP conformation these cut-sites are separated by 6-nt intervals and are at fixed distances from the crRNA 5′-handle [16,21,22,32,35]. This ‘ruler’ mechanism is found in all the different type III systems [14,16,20,34,36,37].

Interestingly, the binding and degradation of the target RNA has been reported to be relatively flexible when it comes to mutations within the protospacer sequence [14,22,35,38]. This low stringency implies that MGE mismatch mutants are still being recognized and neutralized, and hence that escape through mutagenesis is less likely. It is possible that the host can endure the occasional off-target degradation of RNA since the effects would not be as disastrous as for off-target DNA damage. Until now, no self-discrimination mechanism has been found regarding RNA cleavage activity nor has the exact seed region defined. Complementarity between the conserved 5′-handle of the crRNA and the 3′ flanking region of the target RNA (which indicates self-targetting) does not abrogate RNA cleavage [22,36]. Although a potential risk for self-targetting could be RNA generated through transcription of the CRISPR array, it is not expected to be much of a problem since transcription of the CRISPR array typically only occurs in the sense direction, producing RNAs that are identical but not complementary to the crRNA.

### Non-specific ssDNA cleavage

**Figure 3:**
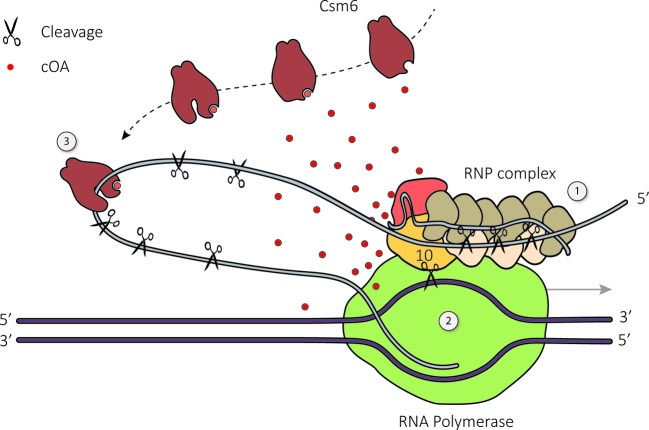
Model for type III RNA and DNA targetting (1) Complementarity between the crRNA and target RNA induces RNA cleavages at fixed 6-nt intervals by the multiple copies of Cas7 present in the RNP backbone. (2) Complementarity between the crRNA and target RNA in combination with non-complementarity between the 5′ crRNA handle and the sequence 3′ of the protospacer region of the target RNA result in the activation of the HD and palm domains of Cas10. The HD domain of Cas10 non-specifically degrades ssDNA that is likely to be available in the transcription bubble caused by RNA polymerase (green) during transcription. The Cas10 palm domain converts ATP to c(OA)s that function as diffusible second messengers. (3) c(OA)s bind to the CARF domains of the Csm6 homodimer. This interaction allosterically activates the sequence non-specific ssRNase activity of its HEPN domains.

In addition to specific target RNA degradation, type III systems were found to facilitate immunity through DNA cleavage [3]. A major breakthrough was the determination that DNA cleavage relies on transcription of the protospacer sequence (Figure 3). On top of that, immunity was only detected when transcription occurred antisense to the protospacer [28,36]. The directional restriction of transcription strongly suggested that a protospacer-containing RNA strand is required for DNA degradation. Several subsequent *in vitro* studies indeed showed that binding of the RNP to a target RNA promotes DNA cleavage [17,35,36]. What came as a surprise was that RNA-induced activation resulted in degradation of both target and non-target DNA sequences [17,35,39]. This indicated that the DNase activity of type III systems is non-specific [35].

Mutational analysis showed that the HD domain of Cas10 is the metal-dependent DNase responsible for this activity [17,35]. Additional *in vitro* work determined that the nuclease activity preferably targets ssDNA, implicating that transcription is required for the production of target RNA as well as for presenting the DNA in a single-stranded form [17]. Upon transcription, RNA polymerase opens the DNA double-helix and uses the template strand to generate an RNA transcript. Meanwhile, the non-template strand is expected to be accessible on the outside of the transcription machinery [40]. This would be in accordance with *in vivo* data revealing that the non-template strand was cleaved and further degraded while the template strand remained intact [36].

Furthermore, it was shown that the three nucleotides complementarity (rPAM) between the crRNA 5′-handle and the 3′ region flanking the RNA protospacer inhibits DNase activity, thereby generating a mechanism to avoid targetting of the host CRISPR array [17,39]. However, this system does not save the host from possible collateral DNA cleavage once activated by a *bona-fide* (invader-derived) target RNA. This issue might be resolved by the spatial–temporal restriction of the DNase activity. Since activation of Cas10 requires the RNP to be bound to the RNA target, the complex is locally restricted to the vicinity of invader RNA transcripts. Moreover, it has been shown that upon cleavage of the RNA target, the cleavage products are released, potentially returning Cas10 to a non-active state [17]. This hypothesis is supported by the detection of increased DNA cleavage upon inactivation of the RNase activity (by mutating the active site of Cas7). Inactivation of the RNase activity causes the target RNA to remain bound to the complex, thereby keeping the Cas10 subunit in an activated state [35].

### Non-specific RNA degradation

In addition to the HD domain mentioned before, Cas10 also contains a palm domain (a domain that has been implicated with cyclase activity in other proteins) harboring a conserved GGDD motif. Although the function of the palm domain in Cas10 has long been enigmatic, recent work has shown that it is involved in the conversion of ATP into a range of cyclic oligoadenylates (c(OA)s) in a metal-dependent way [30,31]. The synthesis of c(OA)s by Cas10 is not constitutive, but requires constant activation by binding of an RNA target to the RNP, and requires non-complementarity between the crRNA 5′-handle and its cognate target RNA [30,31]. It is interesting to note that both the HD-dependent DNase activity and the GGDD-dependent (palm domain) c(OA)s synthesis of Cas10 depend on the non-complementarity of the 5′-handle. This suggests that a common conformational change upon proper target recognition may activate both active sites.

CRISPR-Cas type III systems often harbor genes that encode Csm6 or Csx1 family members [30,41–43]. These homologous proteins contain a C-terminal higher eukaryotes and prokaryotes nucleotide-binding (HEPN) domain and an N-terminal CRISPR-associated Rossman fold (CARF) domain [31,41,44]. A wide range of proteins that belong to the HEPN superfamily have been shown to harbor HEPN-based, metal-independent RNase activity, suggesting that Csm6 or Csx1 also exercise this function [41]. However, neither of the proteins seem to stably associate with the type III effector complex or the adaptation machinery [19,45]. It has been long proposed that the CARF domains of Csm6/Csx1 function as specific sensors for an activation signal [41]. Intriguingly, it was shown that the CARF domain of the homodimeric Csm6 complex can bind the second messengers (c(OA)s) produced by the palm domain of Cas10. Binding of the c(OA)s to Csm6 or Csx1 has been shown to trigger the allosteric activation of its RNase activity [30,31]. This means that recognition of an invader by the type III effector complex not only activates the non-specific nuclease activity by Cas10, but also induces c(OA)s generation by Cas10, which in turn allosterically activates the non-specific ssRNA activity of Csm6. It was determined that *Streptococcus thermophilus* Csm6 (StCsm6) preferentially cleaves at GA or AA. However, the presence of c(OA)s seemed to reduce this apparent preference [30].

Cas10 homologs can produce a range of c(OA)s. For example, StCsm6 is activated by cA_6_ while *Thermus thermophilus* Csm6 (TtCsm6) is activated by cA_4_ [30,31]. This indicates that c(OA)s are second messengers common to type III CRISPR-Cas systems, even though the exact nature of the messenger molecule may differ between systems [30]. Notably, Csm6 and Csx1 proteins from different systems have been shown to exercise RNase activity in the absence of c(OA)s [43–45]. Since this RNase activity is non-specific, this is potentially detrimental for the host. However, the addition of c(OA)s increases the RNase activity dramatically. It is very well possible that the host can withstand the low background RNase activity in the absence of c(OA)s, or that conditions *in vivo* somehow quench this background activity [30,41].

One of the questions that remain to be answered for c(OA)s-induced RNase activity is if, and how, this activity is restrained to invader transcripts. c(OA)s synthesis is regulated through Cas10 activation, and binding of an RNA target induces c(OA)s synthesis while target cleavage by Cas7 terminates c(OA)s synthesis. It is possible that Csm6/Csx1 activity is spatially restricted to the area of c(OA)s production, and that it is temporally restricted by the half-life of c(OA)s-activated Csm6/Csx1 but additional data are required to confirm this hypothesis. Another possibility is that Csm6/Csx1 is not restricted to intruder RNA at all and that it works as a suicide-switch that protects the population, rather than the individual cell, from infection.

## Type III immunity

Type III systems are the only CRISPR-Cas systems that evolved three entirely different nuclease activities. The question remains whether these different nuclease activities are all required to provide efficient protection against invaders. In this section, we will speculate on the origins of type III immunity.

## Immunity primarily against RNA or DNA invaders?

CRISPR systems that adopt two different RNase activities and show spacer adaptation from RNA targets might suggest that RNA invaders are the primary target. The RNase activity of Csm6/Csx1 could ensure complete clearance of the invading RNA, while the Cas7 could facilitate temporal control of this activity. In this scenario, Cas7-mediated RNA cleavage is followed by the release of the RNA degradation fragments from the complex, thereby down-regulating the second messenger (c(OA)s) synthesis. The DNase activity could be accounted for by the fact that some RNA phages replicate through a DNA intermediate. The fact that type III systems can indeed convey resistance to RNA phages supports this hypothesis [22]. A counter argument to this theory is that the CRISPR arrays associated with at least some type III system seem to match DNA phages, rather than RNA phages [17,43]. Additionally, RNA phages have a notoriously high mutation rate which would require swift spacer adaptation by the CRISPR system in order to provide immunity [43].

## Distinguishing lytic from lysogenic infections

One aspect of CRISPR-Cas immunity that is often overlooked is that it can target any intruding MGE, regardless of whether the cargo is harmful or beneficial. Temperate phages can proliferate through two different cycles, a lytic cycle that includes avid expression of its genetic information and results in cell death, and a lysogenic cycle in which the viral DNA is incorporated in the host genome and relies on host proliferation for replication. While the lytic cycle is deadly for the host and should be combated, the lysogenic cycle allows for potentially beneficial genes to be incorporated in the host genome [46,47]. Targetting of these lysogenic infections by CRISPR-Cas could therefore be disadvantageous for the host rather than advantageous [48]. Using its three different nuclease activities, type III systems can tolerate lysogenic infections while responding quickly to lytic onsets, thereby offering the perfect compromise between defense and genetic availability [29,48]. Type III systems that target genes expressed early in the lytic cycle were reported to neutralize the invader using the DNase activity of Cas10 alone [43]. In contrast, targetting of late-expressed genes require the RNase activity of Csm6 to prevent the accumulation of phage RNA [43,49]. This is in accordance with data showing that type III systems can battle lytic infections regardless of whether it targets early or late expressed genes [29]. The RNase activity of Cas7 does not seem to be required for immunity but might function as a switch to lower the DNAse activity of Cas10 after releasing the cleaved target RNA from the complex [19,36].

## Escape mutant targetting

Type III systems are surprisingly flexible regarding mutations in the target sequence [22,35,38]. DNase activity alone is enough to facilitate immunity provided there is full complementarity between the crRNA guide and its RNA target [43]. However, upon introduction of multiple mutations within the protospacer, the RNase activity of Csm6 is required to provide immunity. This suggests that the Cas10 DNase activity is less efficient when the binding affinity between the crRNA and the target RNA is reduced. Csm6/Csx1 is therefore required to prevent accumulation of viral RNA, thereby buying time for the DNase activity of Cas10. This assumption is supported by the fact that a specific type III system not only uses the crRNAs of a coexisting CRISPR-Cas type I-F system, but is also able to neutralize escape mutants from that particular system [24].

## Possible suicide or signaling route

Considering that type III systems rely on two sequence-independent nucleases open up the possibility to use it as a ‘suicide-switch’ or whether it is necessary to cope a high concentration of invader RNA/DNA. It does not seem likely that this is the natural purpose of the system since Cas10 activity is switched off after target RNA degradation and release. Even though it is not currently known how long synthesized c(OA)s will remain present in the cell after Cas10 deactivation, and how long c(OA)s-bound Csm6/Csx1 stays active, thus far, there have been no reports on Csm6/Csx1 causing cell death.

## CRISPR-Cas type VI systems

Unlike any other CRISPR-Cas system, type VI effector proteins have been demonstrated to exclusively cleave RNA targets [50–54]. In fact, type VI effector proteins are endowed with two distinct active sites, both conferring RNase activity: one for pre-crRNA processing and one involved in target RNA degradation, as described below. Type VI effectors are amongst the most divergent CRISPR-Cas proteins studied to date and encompass three subtypes: subtype VI-A (Cas13a/C2c2), VI-B (Cas13b1 and Cas13b2), and VI-C (Cas13c) [13,54]. These Cas13 variants share very low sequence similarity, but can be classified as type VI Cas proteins based on the presence of two conserved HEPN-like RNase domains [51,53,54]. Although these two domains appear to be a conserved feature of Cas13 enzymes and are typically located close to the two terminal ends, their spacing within the protein appears to be unique for each subtype [54].

Currently, there are three published crystal structures of type VI-A Cas13a proteins: Cas13a from *Leptotrichia shahii* (LshCas13a), *Lachnospiraceae bacterium* (LbaCas13a), and *Leptotrichia buccalis* (LbuCas13a) [55–57]. Akin to other Class 2 complexes, the crRNA–Cas13a complex has a bi-lobed architecture, consisting of a nuclease (NUC) lobe and a crRNA recognition (REC) lobe. Despite this similar bi-lobed setup, the overall structure and domains of Cas13a bear very little resemblance to other Class 2 nucleases, such as Cas9 and Cpf1 [58,59]. Instead, the crRNA-bound form of Cas13a adopts a ‘clenched fist’-like structure, with the REC lobe being imperfectly stacked on top of the NUC lobe. The REC lobe has a variable N-terminal domain (NTD) followed by a helical domain (Helical-1), whereas the NUC lobe consists of the two abovementioned HEPN domains (HEPN-1 and HEPN-2) separated by a linker domain (Helical-3). Noteworthy, the HEPN-1 domain is split into two subdomains by another helical domain (Helical-2) [57]. The NTD, Helical-1, and HEPN2 domains form a narrow, positively charged cleft that anchors the 5′ repeat-derived end of the bound crRNA (the 5′-handle), whereas the 3′ end of the crRNA is bound by the Helical-2 domain. The first few nucleotides of the guide portion of the crRNA are buried in the NUC lobe, whereas the central region of the crRNA is in a solvent-exposed state [50,57].

In agreement with the absence of a dedicated pre-crRNA processing nuclease (i.e. Cas6 in type I and III systems), the type VI pre-crRNAs are processed by the Cas13 itself [53]. Typically, the repeats in type VI pre-crRNAs form a bulge-containing stem-looped structure (Figure 1), which appears to be a conserved and essential feature of mature crRNAs in Cas13a effectors, as disruption of the bulge impedes target RNA degradation, whereas pre-crRNA processing remained unaffected [55–57]. Pre-crRNA processing in type VI involves metal-independent cleavages upstream of the stem-loop and does not require a *trans*-activating crRNA (tracrRNA) or host factors [53]. Mutagenesis of positively charged residues in the Helical-1 domain (e.g. Arg^438^ and Lys^441^ in LshCas13a) abrogated pre-crRNA processing, suggesting that this domain is responsible for the formation and binding of the 5′-handle. In agreement with the lack of conservation of some of these residues, the length of the 5′-handle (determined by the cleavage site) can vary between different Cas13 homologs [57,60]. After pre-crRNA processing, the 5′ and 3′ ends of the mature crRNA are anchored within the complex and are ready to engage in target binding.

The presence of two HEPN RNase domains in Cas13 suggested that RNA is the *bona-fide* target substrate for type VI CRISPR-Cas systems. Indeed, a heterologously expressed type VI CRISPR-Cas system in *Escherichia coli* conferred sequence-specific immunity against the lytic phage MS2 (an ssRNA phage that does not have a DNA stage within its life cycle), indicating that Cas13 specifically targets ssRNAs [50,51].

However, it was noted that Cas13-mediated immunity against phage MS2 was accompanied by a growth suppression phenotype. Subsequent biochemical and structural studies unraveled the molecular basis for this phenomenon. It was shown that binding of its cognate target RNA (i.e. a ssRNA complementary to the bound crRNA) converts Cas13 into a sequence non-specific ribonuclease, conferring rapid degradation of neighboring RNAs, an activity often referred to as ‘collateral damage’. Target RNA binding results in the formation of an A-form dsRNA helix (crRNA–target RNA) which causes substantial conformational changes in Cas13a to accommodate the propagating duplex within a positively charged channel in the NUC lobe. Concomitantly, these rearrangements bring the catalytic residues of the two HEPN domains into close proximity, thereby forming a single, composite catalytic site for RNA cleavage [56]. Strikingly, this newly formed catalytic site is formed at a considerable distance from the crRNA–target RNA duplex on the external surface of the protein. Therefore, it was concluded that this highly accessible active site would not only degrade the target RNA in *cis* (provided that the target RNA is long enough to reach the active site), but also conferred promiscuous RNase activity, causing the ‘collateral damage’ to other non-target RNAs in *trans*. Although most RNAs appear to be vulnerable to the promiscuous RNAse activity of Cas13, there are subfamily-specific differences in the nucleotide preferences (e.g. targetting mostly adenosine or uridine-rich RNAs) of these ribonucleases [50,60,61].

These observations gave rise to several questions regarding outcome on the individual cell undergoing type VI immunity, similar to those arising from the observed collateral damage activity by CRISPR-Cas type III immunity described above. For example: is the collateral damage caused by Cas13 aimed at cleaning up local, high concentrations of foreign RNA after binding its target RNA, or does it contribute to thwarting the phage infection by inducing programed cell death or dormancy [62]? Regardless of the answer, it is evident that this activity must be tightly controlled to prevent unwanted spontaneous cellular toxicity. In some type VI-B systems, for example the type VI-associated genes *csx27* and *csx28* appear to have a regulatory role by either repressing or stimulating the collateral activity of Cas13b, respectively [51]. Although the molecular basis for the modulation of Cas13b by Csx27 and Csx28 is currently unknown, it seems to be specific for type VI-B systems, as no clear homologs appear to be associated with type VI-A and VI-C systems. Another layer of control is the requirement of target RNA recognition by Cas13. More specifically, the central, solvent exposed region of the crRNA is thought to initiate nucleation with its cognate target RNA, indicating that Cas13a has a central seed region, opposed to the apically located seed region in type I and type II systems [56]. Mutagenesis of the target RNA complementary to the central seed region of the crRNA demonstrated that two mismatches are enough to prevent Cas13 activation both *in vitro* and *in vivo* and this result was further supported by crystal structure study of target-bound LbuCas13a [50,56]. In addition, target RNA recognition requires the presence protospacer flanking site (PFS) 3′ of the protospacer, although some Cas13b homologs require both 3′ and 5′ PFS, while others appear to have none [50,51,63]. In the case of LshCas13a, for example a non-G PFS motif 3′ of the protospacer is essential for robust RNAi. Speculatively, base pairing of the last nucleotide 5′-handle (cytosine) of the crRNA with G as the 3′ PFS of the target RNA could disrupt the proper positioning of the 5′-handle within LshCas13 (described above) and thereby hamper target recognition/degradation [50,56].

These strict requirements dictating the specificity of Cas13 are perhaps counterbalanced by its unprecedented sensitivity to recognize specific target RNAs that meet these criteria within a heterogeneous population of non-target RNAs. It has been reported that Cas13 can detect target RNAs with femtomolar sensitivity (even down to the attomolar range, when combined with a pre-amplification step), which opened up opportunities to adopt Cas13 for nucleic acid detection applications, for example in disease diagnostics [64,65]. In addition, Cas13 has recently been repurposed to provide sequence-specific immunity toward RNA viruses in plants and knockdown and tracking of specific mRNAs in mammalian cells [66,67]. By fusing a catalytic-dead mutant of Cas13 fused to RNA-modifying enzymes, it is now possible to perform highly specific *in vivo* RNA editing [67]. At last, a nucleic acid detection platform has been devloped which combines nucleases from both the type VI and the type III systems (Cas13 and Csm6, respectively) to increase the signal sensitivity, which illustrates the potential of these RNA-guided RNA cleavage CRISPR-Cas systems to be developed into new applications [65].

Next to the questions surrounding the biological role and promiscuous ribonuclease activity of Cas13, it is also still open for debate how type VI systems acquire spacers. PSI-BLAST and HHpred analyses suggested that some of the subtype VI-A loci have adaptation modules, indicating that these type VI systems can acquire new spacers from RNA bacteriophages directly [53]. However, acquisition from RNA generally requires the involvement of an RT enzyme, as was shown for some type III systems [25,26]. However, with the exception of Lachnospiraceae bacterium MA2020, most type VI systems do not seem to encode a separate RT [53]. Another possibility is that that type VI systems rely on the adaptation modules and CRISPR arrays from other CRISPR-Cas systems residing in the same host, as was recently demonstrated for the type I and III systems in *Marinomonas mediterranea* [24]. Nevertheless, the details underlying spacer acquisition in type VI systems are still waiting to be explored.

## Concluding remarks

Although type III CRISPR-Cas systems were amongst the first to be characterized, only recently are we starting to develop a thorough understanding of how these systems operate. Type III systems are endowed with many different sequence-specific and sequence non-specific (deoxy)ribonuclease activities, which are co-ordinated in a highly organized fashion. The cue for all these activities is the detection and binding of the target RNA, and in this respect, shows conceptual resemblance to the evolutionary distant type VI system (paradoxically, the latest identified CRISPR-Cas system). Both systems seem to confer ‘collateral damage’ toward cellular RNAs, and as outlined in the review, it remains elusive whether this is controlled in a spatial and/or temporal fashion (i.e. to combat phage infection), or whether it kills the infected cell for the sake of the population by inducing programed cell death. The recently discovered involvement of second messenger signaling in conferring this behavior in type III interference was unexpected and intriguing, as it is reminiscent of the usage such molecules in mammalian innate immunity. It also illustrates that there still might be many unexpected aspects in CRISPR-Cas immunity that remain to be discovered.

## Abbreviations

CARF: CRISPR-associated Rossman fold
CRISPR-Cas: clustered regularly interspaced short palindromic repeats, CRISPR-associated genes
crRNA: CRISPR RNA
c(OA): cyclic oligoadenylate
HEPN: higher eukaryotes and prokaryotes nucleotide-binding
MGE: mobile genetic element
NTD: N-terminal domain
NUC: nuclease
PFS: protospacer flanking site
REC: crRNA recognition
RNP: ribonucleoprotein
RT: reverse transcriptase
